# Linking quantitative demands to offshore wind workers’ stress: do personal and job resources matter? A structural equation modelling approach

**DOI:** 10.1186/s12889-018-5808-8

**Published:** 2018-07-31

**Authors:** Janika Mette, Marcial Velasco Garrido, Alexandra M. Preisser, Volker Harth, Stefanie Mache

**Affiliations:** Institute for Occupational and Maritime Medicine, University Medical Centre Hamburg-Eppendorf, Seewartenstr. 10, 20459 Hamburg, Germany

**Keywords:** Offshore wind industry, Quantitative demands, Stress, Psychological detachment from work, Social support, Influence at work

## Abstract

**Background:**

Employees in the offshore wind industry are exposed to various job demands, increasing the workers’ risk of experiencing strain reactions. However, personal and job resources might play a role in the stressor-strain context. The aim of this study was (1) to examine the link between offshore employees’ quantitative demands and stress, and (2) to study the role of personal and job resources (psychological detachment from work, social support, and influence at work) in this stressor-strain relationship.

**Methods:**

Two hundred fifty offshore wind workers responded to an anonymous web-based survey, assessing the workers’ quantitative demands, social support, influence at work, psychological detachment from work, and stress. Descriptive statistical analyses and structural equation modelling were applied to test the hypotheses.

**Results:**

Correlation analyses revealed substantial associations between employees’ quantitative demands, personal and job resources, and stress. Results of structural equation modelling indicated a good fit of the hypothesized model. Quantitative demands were positively related to stress, and psychological detachment from work partially mediated this relationship. Social support was negatively related to stress, while influence at work was not. Neither social support nor influence at work moderated the stressor-strain or stressor-detachment relationship.

**Conclusions:**

The results contribute to the current knowledge on the topic. They can be used to design health promotion interventions aimed at reducing offshore employees’ quantitative demands, fostering their ability to mentally detach from work, and enhancing social support at the offshore workplace.

## Background

The offshore wind industry represents an important element of the green energy revolution in Germany [1]. The relevance of this sector is especially evident in the rising number of offshore wind parks being installed in the North and Baltic Seas [2], as well as in the increasing number of employees working in the branch [1, 3]. The offshore workplace is unlike any other, with offshore employees being confronted with demands unique to this particular setting [4]. Long working hours, hard physical labour, challenging transfer to the offshore installations via ship or helicopter, and periods of absence from home are only some of the factors that make offshore work a demanding and potentially stressful occupation [5]. Being exposed to such stressors at work may increase the workers’ risk of experiencing strain reactions, e.g., stress. However, there is still limited knowledge regarding the working conditions in the German offshore wind industry, and even less is known about the impact of these conditions on offshore workers’ health.

Moreover, the understanding of intervening variables that might influence the relationship between offshore employees’ job demands and strain reactions remains unclear. However, as shown for other occupational samples [6, 7], it can be assumed that the workers’ personal and job resources may help them to deal with their job demands. Recently, various job resources and coping strategies of offshore wind workers have been identified in an interview study [5, 8]. They included the high personal meaning of offshore work, the wide scope of action, the strong comradeship offshore, and the regular work schedule [5]. Moreover, adopting self-determined work behaviour and seeking social support from colleagues were reported as effective coping strategies by offshore wind workers [8].

Given the limited empirical evidence, the present study aimed to close research gaps by addressing various topics. Our purposes were (1) to examine the link between offshore wind workers’ quantitative demands and perceived stress, and (2) to study the role of specific intervening variables in this stressor-strain relationship. These variables included a personal resource (psychological detachment from work) and two job resources (social support and influence at work).

### Linking quantitative demands to offshore wind workers’ stress

In this study, quantitative demands were conceptualized as both extensive and intensive demands inherent to one’s work (e.g., hours of work, pace of work, workload). Stress was regarded as an intra-individual state characterized by high arousal and displeasure, and, thus, conceptualized as a strain reaction.

It has previously been discussed that employees in the German offshore wind industry experience high quantitative demands, e.g., in terms of high work intensity, time pressure, long continuous work periods, and overtime hours [9, 10]. Likewise, time pressure has been named as an organizational hazard for workers in the international offshore wind industries [11]. Regarding the workers’ stress perceptions, a recent qualitative study found German offshore wind workers to describe varying levels of stress in different work situations [8].

In general, empirical evidence suggests that job demands can evoke strain reactions and negatively impact employees’ mental and physical health [12–14], while job resources were found to be linked to positive health effects and to foster employees’ work engagement [15, 16]. Positive associations between quantitative demands and employees’ stress levels have been revealed across different occupations [17–19], including the offshore wind workforce [8]. Precisely, offshore wind employees reported their quantitative job demands to have an impact on their perceived stress levels, fatigue, and sleep quality [8]. Further to this, studies conducted in the offshore oil and gas industries have consistently described adverse effects of job demands (e.g., shift work, high quantitative demands) on offshore workers’ health and stress levels [20–24]. Yet other studies have suggested a positive link between seafarers’ job demands and stress [25], as well as mental and physical fatigue [26]. Such findings are assumed to be applicable to workers in the offshore wind industry to some extent, as the branches share certain similarities (e.g., remote workplaces) [27, 28].

The mechanisms of job demands and resources have been conceptualized in the Job Demands-Resources model (JD-R) model by Bakker and Demerouti [15, 16]. Precisely, the model assumes that job demands constitute aspects of the job that require physical or mental effort, and that high or unfavourable job demands are positively related to the depletion of health (*health impairment process*). Job resources, in contrast, are assumed to reduce job demands and their adverse effects, and to be elemental in increasing employees’ motivation and work engagement (*motivational process*). Drawing upon the empirical evidence and the JD-R model, we hypothesized the following:

- *Hypothesis 1:* Quantitative demands are positively related to offshore workers’ stress.

### The role of personal and job resources in the stressor-strain relationship

Multiple factors likely play a role in the relationship between offshore wind workers’ quantitative demands and stress, such as their social support and influence at work, but also their ability to mentally unwind from work.

#### Psychological detachment from work

Psychological detachment from work is defined as the ability to mentally disengage oneself from work when being away from the workplace, and is considered a strong indicator of psychological recovery [29, 30]. A previous study showed that the ability to mentally detach from work comprised a relevant coping strategy for offshore wind workers [8]. However, some workers also stated difficulties to unwind from work in the evening hours [8].

**Fig. 1:**
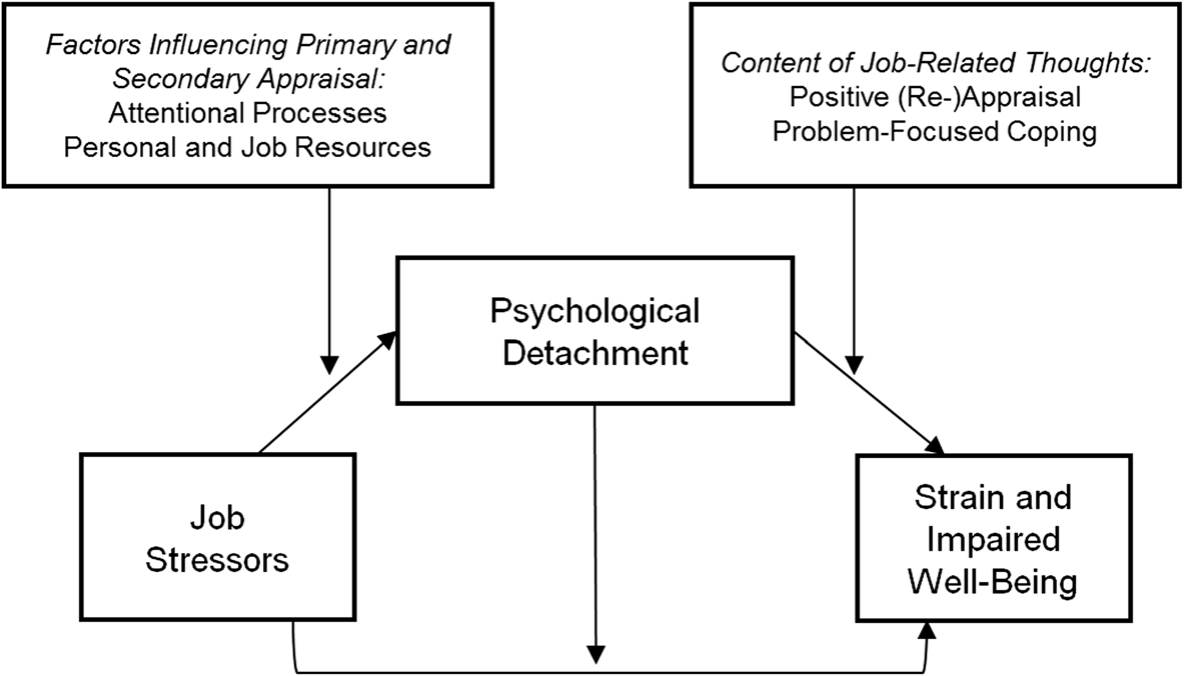
Extended Stressor-Detachment model by Sonnentag and Fritz [29]. Reprinted with permission from [29]

In the Extended Stressor-Detachment model introduced by Sonnentag and Fritz [29], psychological detachment is proposed as a powerful mechanism in the link between stressors and strain reactions (Fig. 1). The model assumes that job stressors impede psychological detachment from work (mainly by increasing negative activation and evoking a state in which it becomes more difficult to detach). Psychological detachment is presented as both a mediator and moderator in the model. The mediation aspect suggests that job stressors impair psychological detachment from work, and, in turn, poor psychological detachment from work directly influences employees’ strain. The moderation aspect assumes that psychological detachment attenuates the effects of job stressors on employees’ strain. A further assumption of the model is that personal and job resources (among other variables) act as moderators, attenuating the effects of job stressors on psychological detachment.

Research studies have provided substantial empirical support for the model by showing negative associations between job stressors (e.g., quantitative demands, workload, time pressure) and psychological detachment from work [29, 31–36]. Moreover, negative links between psychological detachment from work and strain reactions (e.g., need for recovery, fatigue, exhaustion) were revealed [29, 32, 37, 38]. Furthermore, there is evidence of the mediating role of psychological detachment in the stressor-strain relationship [32, 33, 35, 39, 40], but also of its moderating function [37, 38, 41]. However, framing psychological detachment as both a mediator and moderator seems problematic; when the context of job demands influences the chances for psychological detachment to occur, it should rather act as a mediator [33]. Based on this, we conceptualized psychological detachment from work as a mediator in the stressor-strain relationship and proposed the following hypotheses:

- *Hypothesis 2a:* Quantitative demands are negatively related to offshore workers’ psychological detachment from work.
- *Hypothesis 2b:* Psychological detachment from work is negatively related to offshore workers’ stress.
- *Hypothesis 2c:* Psychological detachment from work partially mediates the relationship between offshore workers’ quantitative demands and stress.

#### Social support

Social support can be broadly defined as the availability and quality of helping relationships [42]. In the offshore wind industry, the active pursuit of social support was found to constitute a coping strategy for workers in dealing with their job demands [8]. Positive health effects of social support have also been described for offshore oil and gas workers [22, 23, 43, 44] and seafarers [25]. Moreover, a buffering effect of social support was revealed in the relationship between perceived risks at work and offshore oil and gas workers’ strain [44].

It has been widely proposed that social support reduces strain levels directly and regardless of the stressors’ intensity [45]. Cross-sectional and longitudinal studies have provided evidence for this notion by demonstrating reliable positive associations between social support and employees’ health [45–48]. Apart from its direct effect, a buffering role of social support in the relationship between job demands and employee strain has been theoretically described in the JD-R [15, 16] and Job Demand-Control-Support (JD-C-S) model [49]. This buffering effect has been empirically proven [15, 45], though findings remain inconsistent [50–52]. In addition, in the Extended Stressor-Detachment model, job resources (such as social support) are assumed to moderate the link between job demands and psychological detachment from work [29]. Precisely, high levels of social support are presumed to be conducive to the ability to detach from work, as employees will be confident to get help from others when needed. However, since the Extended Stressor-Detachment model was only introduced recently [29], this moderator effect has yet to be empirically tested. Summarizing the above, we proposed the following hypotheses:

- *Hypothesis 3a:* Social support is negatively related to offshore workers’ stress.
- *Hypothesis 3b:* Social support moderates the relationship between offshore workers’ quantitative demands and stress.
- *Hypothesis 3c:* Social support moderates the relationship between offshore workers’ quantitative demands and psychological detachment from work.

#### Influence at work

Influence at work can be described as employees’ control over their tasks and conduct throughout a workday [53]. Among the German offshore wind workforce, job control was found to comprise an important job resource [5], with increasing job control aiding the ability to cope with demands at work [8]. Furthermore, high levels of job control were found to exhibit positive effects on offshore oil and gas workers’ health [22], and were significantly related to lower levels of mental fatigue in seafarers [26].

Theoretical models, such as the Job Demand-Control (JD-C) [53] and the JD-R model [15, 16], have proposed a positive impact of job control on workers’ health. This has been empirically proven in several studies [18, 54, 55]. The JD-C model assumes that job strain particularly results from a combination of high job demands and low job control [53]. In addition, the JD-R model proposes that job control moderates the negative effects of high job demands on workers’ strain [15, 16]. Although some studies have provided empirical support for this notion [12, 56], evidence remains mixed [57], with reviews suggesting a weak to moderate support for the moderator function of job control [58, 59]. Furthermore, in the Extended Stressor-Detachment model, it is proposed that job resources, such as influence at work, moderate the link between quantitative demands and psychological detachment from work [29]. However, empirical evidence for this effect is still needed. Based on the above, we proposed the following hypotheses:

- *Hypothesis 4a:* Influence at work is negatively related to offshore workers’ stress.
- *Hypothesis 4b:* Influence at work moderates the relationship between offshore workers’ quantitative demands and stress.
- *Hypothesis 4c:* Influence at work moderates the relationship between offshore workers’ quantitative demands and psychological detachment from work.

### Hypothesized model

**Fig. 2:**
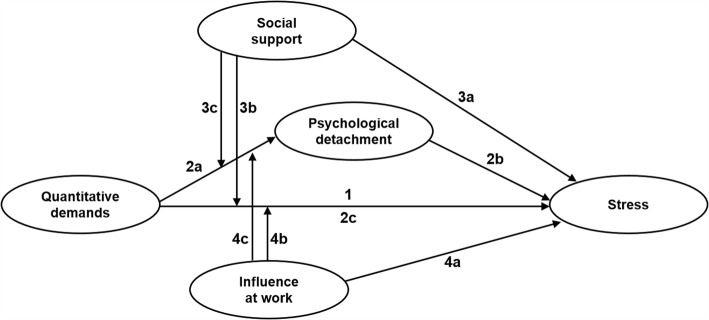
Conceptual model with the hypothesized interrelationships between the variables

The hypothesized interrelationships between the variables are summarized in a conceptual model in Fig. 2.

## Methods

### Study design and participants

The data are based on a sample of workers in the German offshore wind industry. The study was designed as a cross-sectional web-based survey. Data collection took place between September 2016 and January 2017 via a web-based system which permitted secure and anonymous data collection. We used both a German and an English version of the online questionnaire. As inclusion criteria for study participation, offshore workers had to have worked offshore at least 28 days during the last year. An initial internet search was carried out in order to identify offshore companies and service providers. We contacted around 50 small-, medium- and large-sized industry players via telephone and email, sent study information leaflets in German and English to them, and asked them to distribute the information to their employees (e.g., via intranet, newsletters, e-mails, and word-of-mouth promotion). In return for their participation, companies were offered the possibility to receive the study results. We also posted the study information on online platforms for offshore wind workers and sent information leaflets to 40 German occupational physicians who participated in a workshop on occupational medicine in the German offshore wind branch. In addition, we visited an offshore fair (“WindEnergy 2016”) and presented the study at the “Round-table Maritime Safety Partnership” meeting organized by the German Offshore Wind Energy Foundation. Prior to data collection, all respondents were informed about the study aims and data confidentiality, and gave written informed consent. All participants took part in the survey voluntarily.

### Variables and instruments

#### Socio-demographic variables

We used self-constructed items to assess the following socio-demographic variables: gender, age, relationship status, nationality, offshore experience, occupation, work schedule, work shift, project phase of the wind park, and living accommodation.

#### Job demands and resources

Quantitative demands, social support and influence at work were assessed using the same named scales from the Copenhagen Psychosocial Questionnaire (COPSOQ I) [60, 61]. Items were scored on a 5-point Likert scale ranging from 1 (= *always*) to 5 (= *never*). For analysis purposes, item scores were transformed to point values ranging from 0 (minimum) to 100 (maximum) (e.g., for a 5-response category item: 0, 25, 50, 75, and 100). Reliability and validity of the COPSOQ I are good [60, 61].

#### Psychological detachment from work

Psychological detachment from work was assessed with the same named 4-item subscale from the Recovery Experience Questionnaire [34]. The scale ranges from 1 (= *I do not agree at all*) to 5 (= *I fully agree*), with higher scores indicating higher psychological detachment. The scale shows good psychometric properties [34].

#### Stress

Stress symptoms were measured using the 4-item subscale of the COPSOQ II [62]. Items were scored on a 5-point Likert scale ranging from 1 (= *all the time*) to 5 (= *not at all*). Items from the stress scale were transformed to values ranging from 0 (minimum) to 100 (maximum) for data analysis. Research supports the psychometric qualities of the scale [62].

### Statistical analysis

Statistical analyses were performed using IBM® SPSS® Statistics (version 24 [63]) and Analysis of Moment Structures (IBM® SPSS® AMOS™, version 24 [64]). Due to the small amount of missing data in the sample (2.06%) and the non-significant result in Little’s MCAR test (*χ*^*2*^ = 837.59, df = 831, *p* = .43) indicating that data was missing completely at random [65], a single imputation method was applicable [66]. We used the expectation maximization algorithm to achieve a complete dataset [67], a method allowing us to apply bootstrapping procedures in AMOS. Additionally, we ran our analyses by using the Full Information Maximum Likelihood method as an advanced missing data handling technique. The results of both methods were comparable, indicating that there was no bias due to the applied single imputation method. Data was verified for outliers and normality. Although the Shapiro-Wilk-Test indicated that data was not normally distributed, skewness and kurtosis of the variables were mainly beyond the threshold of < 1.0, and histograms showed no substantial deviations from normal distribution. Therefore, we used parametric tests.

We performed descriptive statistical analyses for general characteristics and associations between the variables. To further examine our hypotheses, we used structural equation modelling (SEM). In SEM, the manifest variables (observed items for each scale) acted as indicators of the non-observable latent variables (quantitative demands, stress, psychological detachment from work, social support, and influence at work).

We firstly performed a confirmatory factor analysis (CFA) to test for reliability and validity, and to assess the fit of the measurement model. In the initial measurement model, we specified two error terms of items from the variables psychological detachment and social support to correlate, as suggested by high modification indices. This procedure was theoretically justified in view of the similar content of these items. The assumption of linearity regarding the relationship between the independent and dependent variables was tested and confirmed with deviation from linearity tests. Multicollinearity was rejected for all variables. Skewness and kurtosis of the variables were within the suggested threshold of < 1.0 [68].

In the structural model, we used data collected on age, work schedule, and work experience as control variables by having them regressed on the two endogenous latent variables psychological detachment and stress. We chose Maximum-Likelihood as the method of estimation and used bootstrapping with 2000 iterations to calculate direct and indirect effects when testing the structural model. For assessing mediation, we used the causal steps approach by Baron and Kenny [69]. In addition, we calculated the indirect effect for the mediation path from quantitative demands → psychological detachment → stress in AMOS [70, 71]. To test for moderation, we computed composite factors from the latent variables, standardized them, and computed interaction terms between the independent variable (quantitative demands) and the potential moderators (social support and influence at work).

To evaluate the goodness-of-fit between the hypothesized model and the data, we used *χ*^2^, *χ*^2^/df (ratio of *χ*^2^ to degrees of freedom), Comparative Fit Index (CFI), Root Mean Square Error of Approximation (RMSEA), and Standardized Root Mean Square Residual (SRMR). The following thresholds were used to determine relatively good model fit: CFI ≥ .95, RMSEA ≤ .06, and SRMR ≤ .08 [72]. *P*-values < .05 were considered statistically significant. All provided *p*-values were two-tailed. We used standardized regressions weights (β) to assess the strengths of association between the variables. Based on Cohen’s recommendations [73], β = 0.1 was interpreted as a weak, β = 0.3 as a moderate, and β = 0.5 as a strong association.

## Results

### Sample characteristics

In total, 267 workers completed the relevant scales in the survey and fulfilled the criterion of having worked at least 28 days offshore during the last year. Due to the small size of female workers in the sample (*n* = 13), they were omitted from the analysis, as well as four workers who did not provide information regarding their work schedule (*n* = 2) or gender (*n* = 2). The final sample consisted of 250 male offshore workers.

As shown in Table 1, 118 (47.2%) employees were aged between 30 and 39 years. The large majority of the participants were German (*n* = 221, 89.8%), and claimed to be in a relationship (*n* = 211, 84.7%). Around two-thirds of the workers (*n* = 162, 65.1%) had more than 3 years of offshore experience. Almost half of the workers (*n* = 122, 48.8%) were technicians or mechanics. The majority of the workers (*n* = 215, 86%) had a regular work schedule.

**Table 1:** Participant characteristics

Variables	*Number* (%)
Gender: male	250 (100)
Age (*n* = 250)	
≤ 29 years	46 (18.4)
30 – 39 years	118 (47.2)
40 – 49 years	57 (22.8)
≥ 50 years	29 (11.6)
Relationship status (*n* = 249)	
Single	38 (15.3)
In a relationship	211 (84.7)
Nationality (*n* = 246)	
German	221 (89.8)
Other	25 (10.2)
Offshore experience (*n* = 249)	
≤ 3 years	87 (34.9)
> 3 years	162 (65.1)
Occupation (*n* = 250)	
Management onshore	13 (5.2)
Management offshore / supervisor	78 (31.2)
Technician / mechanic	122 (48.8)
Ship’s / platform crew	14 (5.6)
Research staff / surveyor, medical staff	12 (4.8)
Quality manager / health and safety staff	11 (4.4)
Work schedule (*n* = 250)	
Regular schedule	215 (86.0)
Occasional assignments^a^	35 (14.0)
Work shift (*n* = 250)	
Day shifts only	125 (50.0)
Night shifts only	1 (0.4)
Rotating shifts (day and night shifts)	124 (49.6)
Project phase of wind park (*n* = 249)	
In construction	88 (35.3)
In operation	161 (64.7)
Living accommodation (*n* = 250)	
Offshore – on a platform	87 (34.8)
Offshore – on a hotel ship	64 (25.6)
Offshore – on a construction ship	43 (17.2)
Offshore – in a container on a platform / ship	23 (9.2)
On an island / on the mainland – at a hotel or flat	33 (13.2)

Sample size differs between *n* = 246 and *n* = 250 due to missing data

^a^≥ 28 days offshore during the last year

### Descriptive analysis

Table 2 depicts the characteristics of all variables (means, standard deviations, minimum and maximum values, and Cronbachs Alpha). Reliability was confirmed for all variables (α > .7).

**Table 2:** Characteristics of all variables

Variables		*M*	*SD*	*Min*	*Max*	α
1	Quantitative demands	47.6	19.3	0	100	.72
2	Influence at work	45.4	20.7	0	93.75	.78
3	Social support	71.9	17.9	18.75	100	.81
4	Stress	35.0	20.2	0	81.25	.88
5	Psychological detachment	2.7	0.9	1	5	.92

*M* = Mean, *SD* = Standard deviation, *Min* = Minimum, *Max* = Maximum, *α* = Cronbachs Alpha

Table 3 shows the Pearson correlation coefficients for the variables. Quantitative demands were significantly and positively related to stress, while both job resources (social support and influence at work) were significantly and negatively related to stress. Psychological detachment from work was significantly and negatively related to quantitative demands and stress. Furthermore, it was significantly and positively related to social support, but not significantly related to influence at work.

**Table 3:** Pearson correlation coefficients for all variables

Variables		1	2	3	4	5
1	Quantitative demands	-				
2	Influence at work	-.04	-			
3	Social support	-.24***	.32***	-		
4	Stress	.52***	-.14*	-.41***	-	
5	Psychological detachment	-.42***	-.01	.20**	-.50***	-

Pearson correlation coefficient: *p < .05; **p < .01; ***p < .001

### Structural equation modelling (SEM)

#### Confirmatory factor analysis (CFA)

The characteristics of all variables and items involved in the confirmatory factor analysis (CFA) are depicted in Table 4. The corrected item-total correlations and factor loadings of all items were around or above .50.

**Table 4:** Characteristics of all variables and items involved in the CFA

Variables	*M*	*SD*	*r*_*it*_	factor loadings
Quantitative demands	47.6	19.3		
qd1: *“Do you have to work very fast?”*	52.1	22.4	.53	.61
qd2: *“Is your workload unevenly distributed so it piles up?”*	42.1	25.3	.62	.81
qd3: *“How often do you not have time to complete all your work tasks?”*	44.6	25.2	.57	.74
qd4: *“Do you have to do overtime / extra work?”*	51.5	31.3	.36	.43
Influence at work	45.4	20.7		
infl1: *“Do you have a large degree of influence concerning your work?”*	56.8	25.0	.61	.72
infl2: *“Do you have a say in choosing who you work with?”*	32.9	26.4	.59	.69
infl3: *“Can you influence the amount of work assigned to you?”*	37.6	25.7	.58	.68
Infl4: *“Do you have any influence on what you do at work?”*	54.4	28.9	.58	.68
Social support	71.9	17.9		
supp1: *“How often do you get help and support from your colleagues?”*	76.6	18.5	.52	.48
supp2: *“How often are your colleagues willing to listen to your problems at work?”*	75.0	20.7	.64	.61
supp3: *“How often do you get help and support from your nearest superior?”*	66.0	24.6	.70	.86
supp4: *“How often is your immediate superior willing to listen to your work related problems?”*	70.1	25.5	.65	.82
Stress	35.0	20.2		
stress1: *“How often have you had problems relaxing?”*	34.4	25.1	.71	.77
stress2: *“How often have you been irritable?”*	30.6	22.6	.68	.73
stress3: *“How often have you been tense?”*	37.9	23.5	.77	.86
stress4: *“How often have you been stressed?”*	37.1	23.5	.78	.85
Psychological detachment	2.7	0.9		
* “After an offshore working day…”*				
detach1: *“I forget about work.”*	2.7	1.0	.82	.79
detach2: *“I don’t’ think about work at all.”*	2.4	1.0	.81	.79
detach3: *“I distance myself from my work.”*	2.8	1.0	.82	.89
detach4: *“I get a break from the demands of work.”*	2.9	1.0	.83	.92

*M* = Mean, *SD* = Standard deviation, *r*_*it*_ = Corrected Item-Total Correlation

The fit of the initial measurement model was: *χ*^2^ = 311.931, df = 160, *χ*^2^/df = 1.950, *p* < .001, CFI = .94, RMSEA = .06 [.05–.07], SRMR = .06. After specification of two error terms, the model fit improved. The final measurement model showed a good fit to the empirical data (*χ*^2^ = 224.295, df = 158, *χ*^2^/df = 1.420, *p* < .001, CFI = .97, RMSEA = .04 [.03–.05], SRMR = .06).

Results of the reliability and validity analysis are depicted in Table 5. Reliability was confirmed by showing that composite reliability (CR) was ≥ .70 for all variables. Convergent validity was confirmed by showing that the average variance extracted (AVE) of the variables were around or above .50. Although in particular the variable quantitative demands did not fully reach the recommended threshold for the AVE, we did not consider the deviation to be large enough to justify exclusion of single items, since the scale is well validated. We decided to maintain the scale’s original structure, allowing for comparisons of our results with other research findings. Discriminant validity was proven by showing that the square roots of the average variance extracted (√ AVE) of the variables were greater than the correlations between the variables. We investigated common method variance with Harman’s single factor test. The results showed that 30.7% of the variance in the model was explained by the one-factor solution, providing no indications of substantial common method bias [74].

**Table 5:** Reliability and validity analysis

Variables		CR	AVE	√ AVE	Correlations
1	Quantitative demands	.75	.44	.66	-.47 to .62
2	Influence at work	.78	.48	.69	-.15 to .39
3	Social support	.79	.50	.71	-.42 to .39
4	Stress	.88	.65	.80	-.58 to .62
5	Psychological detachment	.91	.72	.85	-.58 to .22

*CR* = Composite Reliability, *AVE* = Average Variance Extracted, *√ AVE* = Square roots of the Average Variance Extracted, *Correlations* = Correlations between the latent variables

#### Structural model

**Fig. 3:**
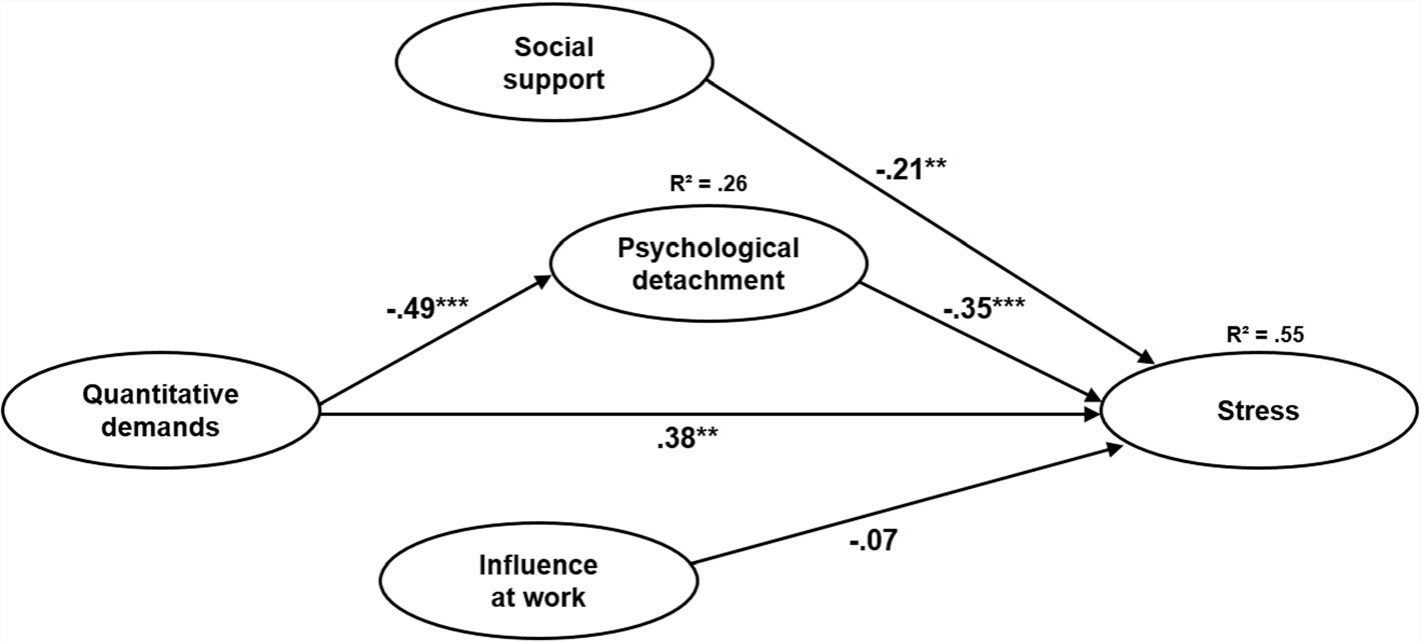
Structural model with standardized path coefficients and squared multiple correlations. χ^2^ = 295.051, df = 205, χ^2^/df = 1.439, *p* < .001, CFI = .96, RMSEA = .04 [.03–.05], SRMR = .06. Standardized path coefficients are presented on the unidirectional arrow paths. R^2^ = squared multiple correlations. Manifest items, residuals, control variables, and correlations between exogenous variables are not displayed. *p < .05; **p < .01; ***p < .001

The structural model showed a good fit to the empirical data, supporting the hypothesized structure (*χ*^2^ = 295.051, df = 205, *χ*^2^/df = 1.439, *p* < .001, CFI = .96, RMSEA = .04 [.03–.05], SRMR = .06). Figure 3 illustrates the model with the standardized path coefficients and information on the explained variance for the endogenous variables psychological detachment from work and stress. The squared multiple correlations (R^2^) were .26 for psychological detachment and .55 for stress. Thus, 26% of the overall variance in psychological detachment, and 55% of the overall variance in stress were explained by the relations proposed in the model.

In support of hypothesis 1, the direct effect of quantitative demands on stress was significant and positive (β = .38 [95% CI = .23; .52], SE = .08, *p* < .01). The effect of social support on stress proved to be significant and negative (β = -.21 [95% CI = -.36; -.09], SE = .07, *p* < .01), so that hypothesis 3a was accepted. Moreover, the effect of influence at work on stress was negative, but not significant (β = -.07 [95% CI = -.20; .06], SE = .07, *p* = .30). Therefore, hypothesis 4a was rejected.

#### Mediation hypothesis

Following the causal steps approach by Baron and Kenny [69], we investigated the direct effect of quantitative demands on stress without controlling for the potential mediator (psychological detachment from work). The effect was significant and positive (C: β = .53 [95% CI = .39; .65], SE = .07, *p* < .01; Table 6). When controlling for the mediator, this effect remained significant, but was decreased (C′: β = .38 [95% CI = .23; .52], SE = .08, *p* < .01). Supporting hypotheses 2a and 2b, there was a significant and negative effect of quantitative demands on psychological detachment (A: β = -.49 [95% CI = -.60; -.35], SE = .06, *p* < .001), and a significant and negative effect of psychological detachment on stress (B: β = -.35 [95% CI = -.49; -.21], SE = .07, *p* < .001). According to the approach by Baron and Kenny, this pattern indicates partial mediation [69].

**Table 6:** Mediation analysis

		Psychological detachment					Stress			
		β	95% CI	SE	*p*		β	95% CI	SE	*p*
Psychological detachment		-	-	-	-	B	-.35	[-.49; -.21]	.07	.001
Quantitative demands	A	-.49	[-.60; -.35]	.06	.001	C	.53	[.39; .65]	.07	.002
						C′	.38	[.23; .52]	.08	.002
Quantitative demands → Psychological detachment						A × B	.17	[.11; .26]	.04	.001

β = Standardized regression weight, 95% CI = 95% Confidence interval [lower bound; upper bound], *SE =* Standard error. *p* = *p*-values: *p < .05; **p < .01; ***p < .001

Moreover, the indirect effect of quantitative demands → psychological detachment → stress (path A x B) was significant and positive (β = .17 [95% CI = .11; .26], SE = .04, *p* < .001), indicating that psychological detachment from work mediated the relationship between quantitative demands and stress. Thus, hypothesis 2c was accepted.

**Fig. 4:**
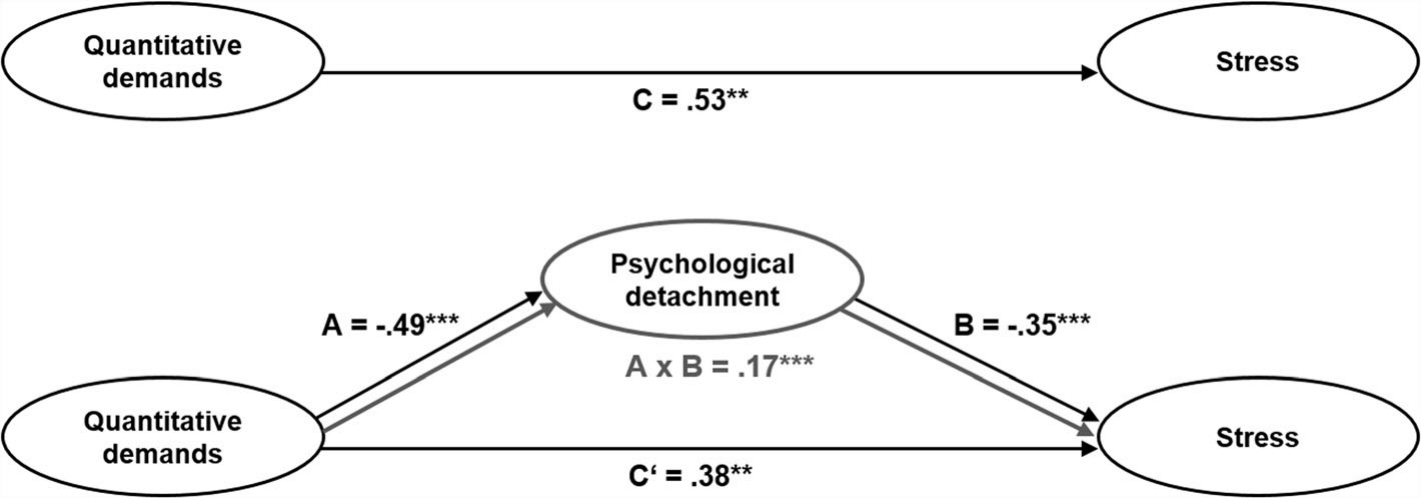
Mediation model for quantitative demands, psychological detachment from work, and stress. Standardized path coefficients are presented on the unidirectional arrow paths. Manifest items, residuals, control variables, and correlations between exogenous variables are not displayed. *p < .05; **p < .01; ***p < .001

Figure 4 shows the mediation model with the standardized path coefficients for the direct pathways (A, B, C), mediated pathway (C′), and indirect pathway (A x B).

#### Moderation hypotheses

We first tested whether social support and influence at work moderated the relationship between quantitative demands and stress. Fit for this model was acceptable (*χ*^2^ = 5.530, df = 4, *χ*^2^/df = 1.383, *p* = .24, CFI = .99, RMSEA = .04 [.00–.11], SRMR = .01). However, neither the interaction term for quantitative demands x social support (β = -.02 [95% CI = -.09; .05], SE = .04, *p* = .69) nor the interaction term for quantitative demands x influence at work were significant (β = -.02 [95% CI = -.11; .05], SE = .04, *p* = .54), indicating that social support and influence at work were not moderating the link between quantitative demands and stress. Thus, hypotheses 3b and 4b were rejected.

We further tested whether social support and influence at work were moderators in the relationship between quantitative demands and psychological detachment from work. The model had an acceptable fit (*χ*^2^ = 5.636, df = 4, *χ*^2^/df = 1.409, *p* = .23, CFI = .99, RMSEA = .04 [.00–.11], SRMR = .01), but results showed that both interaction terms were not significant (quantitative demands x social support: β = -.04 [95% CI = -.15; .07], SE = .06, *p* = .48; quantitative demands x influence at work: β = .05 [95% CI = -.07; .15], SE = .06, *p* = .40). Therefore, hypotheses 3c and 4c were rejected.

## Discussion

The present study set out to examine the link between offshore wind workers’ quantitative demands and stress, and to study the role of personal and job resources (psychological detachment from work, social support, and influence at work) in this stressor-strain context. Our results revealed substantial associations between offshore workers’ quantitative demands, personal and job resources, and stress. In particular, the findings indicated potentially adverse effects of quantitative demands, as well as potentially beneficial effects of social support and psychological detachment from work, on offshore workers’ stress levels.

### Descriptive analysis

To our knowledge, our study is the first in assessing the job demands and resources of workers in the young German offshore wind industry on a quantitative basis. Considering the magnitudes of the variables studied, our results agree with recent qualitative findings regarding the job-related demands and resources of offshore wind workers [5, 8]. However, given the different nature of qualitative and quantitative research, findings from both approaches are not directly comparable.

With respect to offshore employees’ stress perceptions, workers in our study showed higher levels of stress (*M* = 35.0) compared to the available Danish norm sample (*M* = 26.7) [62]. In view of the potentially stressful work environment offshore [75–77], the comparably high levels of stress were to be expected. In line with previous research [5, 8], we found social support to constitute a relevant job resource for the offshore workers, showing a particularly high mean score (*M* = 71.9) when compared with available norm data of samples from German (*M* = 65) [78] and Danish (*M* = 68) [60] workers. The mean score for offshore workers’ quantitative demands (*M* = 47.6) is also comparable to those provided for other norm samples, e.g., for the Danish workforce (*M* = 46.8) [60] or for male German workers in related professions (metal and mechanical engineering, building construction (*M* = 47)) [79]. Consistent with previous findings in which offshore wind workers reported feeling under time pressure [5], we found particularly high mean scores for items concerning employees’ work pace and overtime hours. With respect to offshore workers’ psychological detachment from work, the mean of our sample (*M* = 2.7) is similar, albeit slightly lower, than the mean of the norm sample (*M* = 3.0) [34] and those found for other occupational groups (*M* between 3.2 and 3.7) [36, 38, 80]. This agrees with a previous qualitative finding, suggesting that some offshore wind workers struggled to mentally detach from work [8].

Overall, the correlation analyses revealed that the interrelationships between employees’ quantitative demands, job resources, and stress were in the expected directions. Consistent with the JD-R model’s assumptions [15, 16] and with empirical studies in the offshore oil and gas industry [20, 22–24, 81], we found positive associations between employees’ quantitative demands and stress, and negative associations between their job resources and stress.

### Structural equation modelling (SEM)

**Fig. 5:**
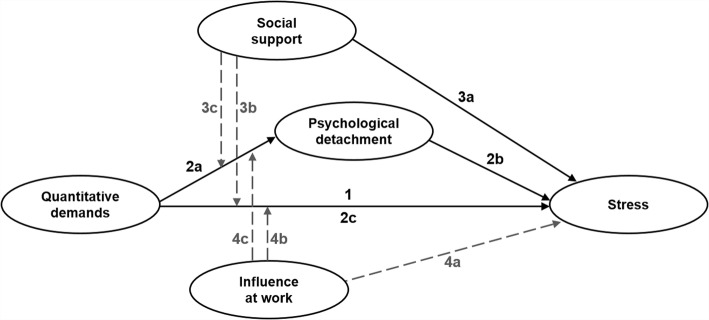
Conceptual model with the accepted and rejected hypotheses. Rejected hypotheses are shown in grey and in dotted arrows

The results of the SEM indicated a good fit of our conceptual model, suggesting that the predictive relationships in the model were compatible with the empirical data. Several hypotheses were accepted on the basis of the SEM results, though not all (Fig. 5).

The evaluation of the path coefficients showed that quantitative demands were positively related to employees’ perceived stress (hypothesis 1), with the association being moderate to strong. This result is in accordance with the propositions of the Extended Stressor-Detachment model [29] and with previous research conducted among offshore wind workers [8], offshore oil and gas workers [22, 23], and the general workforce [17–19, 25]. In particular, our finding supports qualitative research results indicating positive associations between offshore wind workers’ job demands (e.g., time pressure, workload) and stress [8].

Furthermore, quantitative demands were strongly and negatively related to psychological detachment from work (hypothesis 2a) which is consistent with previous research [31, 32, 82] and with the assumptions of the Extended Stressor-Detachment model [29]. The result reinforces the notion that specific characteristics of offshore work (e.g., high workloads and responsibilities) may be associated with employees’ difficulties to unwind from work [8].

Psychological detachment from work, in turn, was found to be negatively related to employees’ stress (hypothesis 2b), with a predictive value suggesting that increased levels of detachment may be beneficial in reducing workers’ stress levels. This association was moderate to strong. The result highlights the importance of psychological detachment as a personal resource and relevant coping strategy for offshore wind workers [8]. Moreover, it is consistent with the assumptions of the Extended Stressor-Detachment model [29] and with studies showing negative links between psychological detachment from work and strain in other occupational groups [29, 32, 37, 38].

A key finding of our study is that psychological detachment from work partially mediated the relationship between quantitative demands and stress (hypothesis 2c). On the basis of this, one might conclude that not only high quantitative demands, but also a resulting lack of psychological detachment from work, may lead to increased stress levels among offshore employees. This further highlights the integral role of psychological detachment for offshore workers. The result agrees with the assumptions of the Extended Stressor-Detachment model [29] and supports studies showing psychological detachment to act as a mediator in the stressor-strain context [32, 33, 35, 39, 40].

Interestingly, while social support was negatively related to stress (hypothesis 3a), influence at work was not (hypothesis 4a). The negative association between social support and stress was weak to moderate. The result is consistent with the JD-R model [15, 16] and agrees with a range of studies that have consistently found positive health effects of social support for the general work population [45–48] and for offshore oil and gas workers [22, 23, 43, 44]. The finding indicates that increased levels of social support may be helpful in reducing offshore workers’ stress, which seems reasonable: workers who perceive having high levels of social support might be more likely to seek help from colleagues, thereby increasing their capacity to cope with stressful work situations.

The non-significant effect of influence at work on stress is in contradiction with our hypothesis and with the assumptions of theoretical models, such as the JD-C model [53] and JD-R model [15, 16]. Moreover, it contradicts previous research that identified high levels of job control as a job resource for offshore wind workers [5, 8], and found job control to be related to lower strain levels in offshore oil and gas workers [22] and seafarers [26]. Overall, it appears that the workers in our sample did not perceive influence at work as a particularly strong job resource. This is also reflected in the variable’s mean score (*M* = 45.4), being similar to those provided for the general German work population (*M* = 47) [78] and for related groups of male German workers (e.g., construction building: *M* = 44) [79]. However, the non-significant effect could also be related to the choice of items used for assessing influence at work: the items measured employees’ general degree of influence, their influence on whom to work with, on the amount of work, and on their work tasks. These aspects, although important, may not be decisive in reducing offshore workers’ stress. In contrast, other aspects of influence at work (e.g., regarding the work environment) may indeed have a predictive value for their stress levels. This seems plausible, since offshore workers were previously found to relate stress to specific aspects of their work environment (e.g., unpredictable weather, accident risks) [8]. Moreover, the impact of influence at work on stress may vary between different groups of offshore staff. Previously, different work groups offshore were found to face different stressors, affecting their stress levels differently [83].

Our results did not support the moderating role of social support and influence at work in the relationships between quantitative demands and stress (hypotheses 3b and 4b), or between quantitative demands and psychological detachment (hypotheses 3c and 4c), respectively. These findings suggest that – regardless of offshore employees’ levels of social support and influence at work – quantitative demands are positively related to stress and negatively related to psychological detachment from work. Moderator effects of social support and job control have rarely been examined in the offshore setting. Although a previous study found social support to buffer the link between offshore oil and gas workers’ perceived risk and strain, this same study did not find buffering effects of social support for several other relationships [44]. The general evidence on the buffering effects of social support and influence at work in the stressor-strain context is mixed [50, 52, 57, 58]. Some studies, in fact, did not find moderating effects of social support [50–52] and influence at work [18, 84]. Moreover, previous research has casted doubt on the existing evidence by highlighting methodological issues and a potential publication bias [57]. Empirical evidence is also scarce regarding the moderating effects of job resources in the relationship between quantitative demands and psychological detachment from work. Our results suggest that social support and influence at work do not mitigate this relationship, thereby contradicting the assumption of the Extended Stressor-Detachment model [29].

We are, however, unable to determine whether both job resources truly do not moderate the investigated relationships, or whether the non-significant results are due to methodological issues. Buffering effects of job resources are more likely to be expected when the resources adequately match the job demands [58]. Thus, there might have been a mismatch between the quantitative demands and the aspects of influence at work and social support assessed in our study. Another methodical concern refers to the rather broad conceptualization of the variables in our study. In past research, studies measuring a *specific* job demand and a *specific* corresponding job resource were more likely to show moderating effects [58].

### Strengths and limitations

Our study has several strengths. In particular, the study is unique in its focus on the occupational health of workers in the rapidly growing German offshore wind industry. So far, there are no comparable investigations that have provided insights into the links between offshore wind workers’ demands and strain reactions on a quantitative basis. Furthermore, our results shed light on the role of relevant personal and job resources (e.g., psychological detachment from work, social support) in the stressor-strain context, thereby expanding the scientific evidence. An important strength of our study lies in the use of SEM as an advanced statistical modelling technique. Combining regression analysis, path analysis, and confirmatory factor analysis, SEM is assumed to have several advantages compared to other statistical techniques. For example, it allows to simultaneously examine all study variables and interrelations of independent variables in one model. Moreover, it permits to adjust for the presence of measurement error and thereby promotes more useful data analyses [85]. Another strength of our study is the use of well-validated instruments that have previously shown strong validity and high internal consistency. Moreover, we performed thorough reliability and validity checks prior to testing our models, which helped in fostering the interpretability of our results.

Our study is not without its limitations, however. Due to the cross-sectional study design, causal inferences cannot be drawn from our results, and reverse causality in the relationships between the variables cannot be completely ruled out. However, at least with regard to the link between quantitative demands and stress, sound evidence suggests that job demands predict changes in strain reactions over time [13]. As a general concern, the application of a mediation model to cross-sectional data assumes that the causes of the variables are instantaneous, and that the magnitude of the effects is independent from the length of time elapsing between the measurements of the variables [86]. This consideration may bias parameter estimation and result in over- or underestimation of true effects [87]. Moreover, data in our study was assessed solely by self-report measures, and participants were recruited in many different ways, including via online platforms. This procedure is helpful in reaching as many participants as possible from a hard-to-reach population, but also increases the risk of self-selection. In addition, the strategy impeded the calculation of a response rate, so that a potential non-response bias in our data was not assessable. The existence of such biases would restrict generalizations of our results. In general, the representativeness of our sample for the total of employees working in German offshore wind parks (*n* = 7600 [88]) is difficult to assess, since relatively few is known about the characteristics of this target population. At least, recent data gathered among works councils in the German on- and offshore wind branch suggests that characteristics of our sample, e.g., the age structure and gender distribution, indeed reflect the actual employment structures in the branch [89]. This has also been reinforced by experts from the German offshore wind sector (e.g., managers of offshore wind farm operators, occupational physicians, and experts from trade associations and maritime societies) with whom we thoroughly discussed our sample’s characteristics.

### Implications

By providing quantitative research results, our study can contribute to a more in-depth discussion and scientific examination of the working conditions and health of workers in the growing German offshore wind industry. Yet, additional research in the area is warranted to verify our findings. Longitudinal studies should be conducted to examine short and long-term dynamics between the variables and gain evidence on the causality of the proposed interrelationships. Such studies should incorporate further personal and job resources of potential relevance, e.g., offshore employees’ workplace commitment and possibilities for development at work. In addition, employees’ health behaviours and their impact on the workers’ health is another topic that merits exploration.

Practical implications can also be derived from our results. Since a substantial association between offshore employees’ quantitative demands and stress was revealed, this indicates a need for health promotion interventions to reduce the workers’ quantitative demands and thereby diminish potential negative health effects. Furthermore, offshore employees should be encouraged to adhere to their work hours and avoid overtime work. From an organizational standpoint, efforts should also be made to enhance offshore workers’ psychological detachment from work and social support, since these resources may be particularly beneficial to reduce employees’ perceived stress. Environmental measures to foster psychological detachment from work may include, e.g., enlarging the spatial distance between offshore employees’ workplaces and living accommodations, and providing the workers with sufficient quiet areas to unwind from work. Considering the restricted spaces offshore, these aspects should be addressed in the planning of new wind parks. On a behavioural level, offshore workers should learn about the importance of mentally detaching from work as well as about the adverse consequences of poor psychological detachment. Participation in recreational activities (e.g., social meetings, sport events) could also foster employees’ psychological detachment from work. The promotion of social gatherings seems to be particularly rewarding in two ways: not only may such events help the workers to mentally unwind from work, but they may also increase the social support and sense of community at the offshore workplace.

## Conclusions

Novel understanding has been provided regarding the interrelationships between offshore wind employees’ quantitative demands, personal and job resources, and perceived stress. The findings can be used to design health promotion interventions to reduce offshore employees’ quantitative demands, foster their ability to mentally detach from work, and enhance social support at the offshore workplace. This may reduce the workers’ stress levels and improve the work environment offshore. From a preventive point of view, such interventions could contribute to sustaining offshore workers’ health in the long term.
